# Mathematics anxiety reduces default mode network deactivation in response to numerical tasks

**DOI:** 10.3389/fnhum.2015.00202

**Published:** 2015-04-21

**Authors:** Belinda Pletzer, Martin Kronbichler, Hans-Christoph Nuerk, Hubert H. Kerschbaum

**Affiliations:** ^1^Department of Cell Biology, University of SalzburgSalzburg, Austria; ^2^Department of Psychology, University of SalzburgSalzburg, Austria; ^3^Centre for Cognitive Neuroscience, University of SalzburgSalzburg, Austria; ^4^Neuroscience Institute and Department of Neurology, Christian Doppler Clinic, Paracelsus Medical UniversitySalzburg, Austria; ^5^Department of Psychology, IWM-Knowledge Media Research Center, University of TuebingenTuebingen, Germany

**Keywords:** mathematics anxiety, number processing, default mode network, processing efficiency, inhibition, working memory, BOLD-response

## Abstract

Mathematics anxiety is negatively related to mathematics performance, thereby threatening the professional success. Preoccupation with the emotional content of the stimuli may consume working memory resources, which may be reflected in decreased deactivation of areas associated with the default mode network (DMN) activated during self-referential and emotional processing. The common problem is that math anxiety is usually associated with poor math performance, so that any group differences are difficult to interpret. Here we compared the BOLD-response of 18 participants with high (HMAs) and 18 participants with low mathematics anxiety (LMAs) matched for their mathematical performance to two numerical tasks (number comparison, number bisection). During both tasks, we found stronger deactivation within the DMN in LMAs compared to HMAs, while BOLD-response in task-related activation areas did not differ between HMAs and LMAs. The difference in DMN deactivation between the HMA and LMA group was more pronounced in stimuli with additional requirement on inhibitory functions, but did not differ between number magnitude processing and arithmetic fact retrieval.

## Introduction

Mathematics anxiety is a negative emotional response toward number manipulation, characterized by high arousal and physiological reactivity and resulting in avoidance of situations requiring mathematical reasoning (Richards and Suinn, 1972; Dew et al., 1984). The prevalence of mathematics anxiety is high (up to 60%) in college students (Betz, 1978). Mathematics anxiety has been observed to negatively influence mathematical performance as measured by standardized achievement tests (Dew et al., 1984; Cooper and Robinson, 1989; Engelhard, 1990; Hembree, 1990; Musch and Broder, 1999; Miller and Bichsel, 2004), although the empirical relationship between mathematics anxiety and mathematical performance is of moderate strength (see Ashcraft, 2002 for a review; view DiLullo, 1997 for a meta-analysis). Mathematical skills are a far better predictor of mathematical performance than mathematics anxiety (Musch and Broder, 1999), since not all individuals with high mathematics anxiety (HMAs) are equally impaired (Lyons and Beilock, 2011, 2012). Nevertheless, performance of HMAs may not reflect their actual skill level, thereby threatening their professional success. Consequently, the assessment of mathematical skills in HMAs is always confounded with their high anxiety. Therefore, it is of uttermost importance to understand the neurocognitive mechanisms of mathematics anxiety without confounding them with mathematics performance. However, as people with poor mathematical skills are at greater risk to develop mathematics anxiety (Wang et al., 2014), it is difficult to investigate the neural correlates of mathematics anxiety without confounds related to mathematics performance.

By now a small number of fMRI studies established that performance deficits in HMAs are not related to activation in parietal areas involved in number processing, indicating that mathematics anxiety is not merely a consequence of negative experiences in individuals with poor mathematical skills (Lyons and Beilock, 2011, 2012). Note however, that mathematics anxiety in children was related to reduced activity in posterior parietal areas involved in number processing (Young et al., 2012). Performance deficits in HMAs rather relate to activity in frontal regions involved in the reappraisal of negative emotions as well as in subcortical regions related to motivational factors (Lyons and Beilock, 2011). Consequently, HMAs may successfully overcome their performance deficits by learning to control their negative emotional response. Nevertheless, such intervention strategies may be associated with significant working load costs for HMAs during number manipulation.

A recent study demonstrated that people with a general disposition for anxiety are at greater risk to develop mathematics anxiety (Wang et al., 2014). Therefore, it seems plausible to assume that the neural correlates of mathematics anxiety can be integrated into the framework of the general neural correlates of anxiety. It has been demonstrated that anxiety in general impairs cognitive efficiency (Eysenck et al., 2007; Ansari and Derakshan, 2011). Aversive stimuli draw attention in highly anxious individuals, resulting in a reduced amount of working memory resources left to allocate toward the cognitive task. As for the case of mathematics anxiety, the aversive stimuli, i.e., numbers, are bound to the task itself, it can be assumed that HMAs are preoccupied with the emotional content of the stimuli, leaving a reduced amount of working memory resources for their cognitive manipulation. Consequently, mathematics anxiety should impair mathematics performance more strongly in tasks with high cognitive load. Indeed the negative relationship between mathematics anxiety and mathematical performance is modulated by task difficulty (Hopko et al., 1998, 2003; Ashcraft, 2002; Cates and Rhymer, 2003), time pressure (Faust et al., 1996; Tsui and Maziocco, 2007), performance pressure and experience with mathematical problems (Morris et al., 1978). Inhibitory functions of working memory are impaired in anxious individuals in particular (Derakshan et al., 2009; Wieser et al., 2009); therefore, tasks requiring inhibitory functions/cognitive control should be particularly affected also in the case of mathematics anxiety. However, even though a lack of inhibitory control in math anxious individuals has previously been proposed (Hopko et al., 1998, 2003), the specific impairment of inhibitory functions in number processing has to our knowledge not been tested previously. However, the involvement of cognitive control in numerical tasks has been shown in many papers in recent years; for instance, it has been discussed extensively for various number types, like two-digit numbers, fraction, decimal numbers or negative numbers (Notebaert and Verguts, 2008; Verguts and Notebaert, 2009; Macizo and Herrera, 2011, 2013; Huber et al., 2013, 2014; Moeller et al., 2013).

Processing efficiency has been associated with increased activation in brain areas involved in attentional/cognitive control (e.g., the dorsolateral prefrontal cortex—DLPFC) and increased deactivation in the task-related default mode network (DMN) (Fales et al., 2008). Reduced activity of the DLPFC has been demonstrated during number processing in a group of highly math anxious children (Young et al., 2012). The DMN includes the medial prefrontal cortex (mPFC), precuneus and posterior cingulate cortex (PCC) and the inferior parietal and temporal lobules. Among a variety of unspecific, cognitively undemanding functions, the DMN supports introspective social, self-referential, and emotion processing (Maddock, 1999; Gusnard and Raichle, 2001; Mazoyer et al., 2001; Greicius et al., 2003). Consequently, it is active during the resting state and deactivates during goal-directed behavior toward external stimuli. Brain regions, particularly involved in emotional processing, are the PCC (processing of emotionally salient stimuli; Maddock, 1999), and the mPFC (mediating the interplay of emotional and cognitive functions; (Gusnard et al., 2001; Raichle et al., 2001; Simpson et al., 2001). Deactivation in DMN areas is indirectly proportional to an increase in activation in cognitive control areas. Consequently, task induced deactivation within the DMN is a positive predictor of performance (Anticevic et al., 2010; Sambataro et al., 2010). If a high proportion of HMAs working memory resources are allocated toward the control of negative emotions, DMN areas of HMAs should be more strongly activated, i.e., less deactivated, during number processing. In line with this idea it has been demonstrated that anxious individuals in general tend to show higher DMN activation in the presence of aversive stimuli during rest (Simpson et al., 2001; Zald and Pardo, 2002). Thereby, less resources remain for the performance of the task itself, resulting in insufficient cognitive control. Consequently, an impairment of DMN deactivation should particularly affect tasks requiring inhibitory functions/cognitive control.

To test the hypothesis that processing efficiency in HMAs is limited because they do not deactivate DMN regions, the present study compares DMN deactivation during number processing between HMAs and individuals with low mathematics anxiety (LMAs). To dissociate processing efficiency from performance deficits, unlike in previous fMRI studies of mathematics anxiety, the two groups were matched for their performance in classical curricular mathematical tasks before entering the fMRI study. We assumed that in order to reach equal performance participants with high math anxiety need more effort to (cognitively) control their anxiety and to focus on the math task at hand. Concluding from the results on general anxiety, this additional effort to regulate their negative emotional response in HMAs should be reflected in higher activation/less deactivation of the DMN. Participants completed a number comparison task, for which behavioral effects of mathematics anxiety have previously been demonstrated (Maloney et al., 2011), as well as a number bisection task. To control for the mathematics specificity of effects, as well as for potential differences in general intelligence, the participants also completed a mental rotation task, testing their spatial abilities and spatial processing, and a verbal reasoning task, testing their verbal abilities and verbal processing. In number comparison participants had to decide which of two vertically aligned two-digit numbers was the larger one (e.g., 27 vs. 63). In number bisection, participants had to decide whether the middle of three two-digit numbers in a row was the correct mean of the outer two numbers (e.g., 14_16_18).

These tasks were chosen, since, as compared to addition or subtraction problems, they are not traditional curricular tasks and were relatively unfamiliar to both HMAs and LMAs, so that differential instruction effects were minimized. Furthermore, the number comparison task allows to manipulate the requirements on inhibitory functions/cognitive control, while the number bisection task allows for the comparison of different types of number processing as described by Dehaene et al. (2003). In number comparison, place-value integration was examined by the compatibility effect. Items were unit-decade compatible if the larger number (determined by the larger decade digit) contained the larger unit-digit (e.g., 47 vs. 23; 4 > 2 and 7 > 3). They were unit-decade incompatible when the larger number (with the larger decade digit) contained the smaller unit digit (e.g., 43 vs. 27; 4 > 2, but 3 < 7). Importantly, unit-decade incompatible items as compared to compatible items require the inhibition of irrelevant unit-digit information (Nuerk et al., 2001, 2011). A strong involvement of cognitive control processes in incompatible items has previously been demonstrated (Notebaert and Verguts, 2008; Macizo and Herrera, 2011, 2013; Moeller et al., 2013). As we assume that inhibitory functions are particularly impaired in HMAs, their processing efficiency may be particularly poor as compared to LMAs in incompatible items.

In number bisection, triplets were considered multiplicative if they were part of a multiplication series (e.g., 14_16_18) and non-multiplicative otherwise (e.g., 13_15_17). Multiplicative items may be solved by direct arithmetic fact retrieval, while non-multiplicative items—like number comparison—require number magnitude processing. Previous evidence suggests that these two processes rely on different representational and neural systems (Nuerk et al., 2002; Wood et al., 2008; Pletzer et al., 2011). While the retrieval of math facts is more strongly automatized than the series of magnitude manipulations required solving non-multiplicative items, neither multiplicative nor non-multiplicative items require inhibitory functions in the number bisection task. It is essential that unlike in a multiplication task, no arithmetic fact has to be retrieved in the number bisection task, but that rather retrieval of multiplication tables can help to solve this magnitude judgment task (in which the magnitude of the middle number has to be judged in relation to the magnitudes of the outer numbers).

In summary, the present study was designed to examine the neural correlates of mathematics anxiety and integrate them into the framework of the general neural correlates of anxiety. Importantly, the present study seeks to investigate the effects of mathematics anxiety on processing efficiency independent of its negative effects on mathematics performance, especially since individuals with already poor mathematical skills are at greater risk to develop mathematics anxiety (Wang et al., 2014). In that respect we hypothesize that LMAs show stronger DMN deactivation than HMAs and that this difference is specific to mathematics, independent of the type of math task, but increases with increasing demands on inhibitory control functions.

## Material and methods

### Participants

Eighteen participants (13 female, 5 male) with low and 18 participants (12 female, 6 male) with HMA scores were selected for the fMRI study. In order to ensure fMRI safety for the participants, they were screened by self-report for no presence of metal in the body, no permanent make-up, no tattoos, no pregnancy and no claustrophobia. All subjects gave their informed written consent to participate in the study. All methods conform to the Code of Ethics of the World Medical Association (Declaration of Helsinki).

### Mathematics anxiety

Mathematics anxiety was assessed by the MARS30-brief (Mathematics Anxiety Rating Scale) by Suinn and Winston (2003), a reliable questionnaire which consists of 30 items. Each item describes a situation involving mathematics. For each item participants reported their level of anxiety associated with the described situation on a 5-point Likert-scale. Thus, item scores ranged from 0 to 4. For each participant the total MARS score was calculated as the sum of all 30 item-scores.

Additionally, participants completed the trait version of the State-Trait-Anxiety Inventory (STAI).

### Mathematics performance

We used four different tasks in a paper and pencil design to evaluate mathematical skills. Two-digit subtractions and one-digit multiplications were presented as verification tasks, for which participants had to decide whether a given solution probe was correct or incorrect. These tasks evaluate rather basic arithmetic abilities and differentiate between number magnitude processing, which is involved in subtractions, and verbal number processing, which is involved in multiplications (see e.g., Dehaene et al., 2003). The more complex divisions and percentages were presented as production tasks, for which participants had to calculate the correct result. Participants had a limited time period to solve each task. Thereby the number of items per task was chosen high enough, that even the most gifted participants were unable to solve all items. For each task operation speed and operation power were calculated. Operation speed was the ratio of completed items among all attempted items. Operation power was the ratio of correctly answered items among completed items. Since they were not normally distributed all speed and power ratios were arcsine transformed. By confirmatory factor analysis we were able to reduce the number of mathematical ability measures to three major components (for details see Pletzer et al., 2010). A Speed measure was calculated as the mean of the four arcsine transformed speed ratios. Measures of Verification Power and Production Power were calculated as the mean of the power ratios of the two verification and production tasks, respectively.

### Procedure

In order to identify individuals who would subsequently participate in the neuroimaging study, we evaluated mathematics anxiety and mathematics performance (see below for a detailed description of tasks) in a large sample of 127 participants (mean age: 22 years, range: 19–40 years) recruited in introductory psychology courses and via adverts. Mathematics anxiety was assessed first and on a separate day from mathematics performance in order to exclude any interference effects of performance testing on anxiety ratings. We identified a weak negative relationship between mathematics anxiety and mathematics performance (Supplementary Figure 1). The mean mathematics anxiety measure was 36.95 ± 14.40. Low and HMA scores were defined as lying in the first (total MARS score < 27.23) or forth quartile (total MARS score > 46.47), respectively. Thirty three participants were found to have low MARS scores, 28 participants had high MARS scores. From each group we were able to select 22 participants, who were matched one-to-one in the three mathematics performance scores to the participants of the other group. They were contacted and screened for their interest in and eligibility for the fMRI study. Eighteen from each group agreed to participate (see Participants). The drop-out of four participants in each group did not affect the mean mathematics performance measures. Thus, mathematics performance was matched between the two groups selected for the fMRI study (Table 1) in order to evaluate processing efficiency in the absence of performance deficits.

**Table 1:** **Characteristics of individuals with high and low mathematics anxiety**.

**Mathematics anxiety**	**Age**	**MARS**	**Speed**	**PowerP**	**PowerV**
Low	23.11 ± 2.31	19.38 ± 5.39	1.58 ± 0.23	2.24 ± 0.53	2.83 ± 0.09
High	25.14 ± 5.20	54.00 ± 9.18	1.54 ± 0.21	2.24 ± 0.40	2.77 ± 0.11

### Neuroimaging tasks

All stimuli were presented on an MR-compatible back-projection screen using Presentation Software (version 0.71, 2009, Neurobehavioral Systems Inc., Albany, CA, USA). Participants completed the following tasks, in the order as described below:

#### Non-numerical tasks

Participants completed a mental rotation task and a verbal reasoning task to control for the mathematics specificity of effects. However, due to scanning time limits, only a short session was available for each task. During 10 mental rotation items participants had to decide, which of four possible dies matched a probe die with different symbols on each side. Dies could be rotated or flipped or flipped and rotated. Stimuli were presented for 12 s with 6 s inter-stimulus interval, resulting in a total of 4.5 min for this functional run. During 10 verbal reasoning items, participants had to decide, which of four possible words matched an analogy (e.g., wood: trees = lawn: ?). Stimuli were presented for 7 s with 3.5 s inter-stimulus interval, resulting in a total of 4.1 min for this functional run.

#### Number comparison task

In a set of 150 items participants had to choose the larger of two two-digit numbers presented above each other. In half of the items the upper number was larger and in the other half the lower number was larger. Numbers ranged from 21 to 98. In 30 items (within decade/WD items) the two numbers contained the same decade-digit. These items were included in order to not allow participants to fully ignore unit digits in their comparisons. In the remaining 120 items (non-WD items) all four digits were different. Half were *unit-decade compatible* (C), i.e., the smaller number contained the smaller unit digit (e.g., 23_68, 2 < 3 and 6 < 8). The rest was *unit-decade incompatible* (I), i.e., the smaller number contained the larger unit digit (e.g., 28_63, 2 < 6 but 8 > 3). Additionally, a control item consisting of four pound keys (##_##) instead of numbers was presented 30 times (null event). Each item was presented for 2 s and followed by a 1 s inter-stimulus interval, resulting in a total of 9 min for this functional run.

#### Number bisection task

In a set of 160 items participants had to decide, whether the middle of three two-digit-numbers was the correct mean of left and right number. Numbers were displayed in a row (the smallest number on the left, the largest number on the right) and separated by an underline character (e.g., 12_15_18). In half of the items the middle number was the correct mean of left and right number (correctly bisected items), in the other half the middle number was smaller or larger than the correct mean of left and right number (not correctly bisected), but always lay within the range of left and right number. In all items the correct mean of left and right number was an integer. Correctly bisected (CB) items were considered *multiplicative*, if the triplet was part of a multiplication series (e.g., 12_15_18), and *non-multiplicative* otherwise (e.g., 13_16_19). Additionally a control item consisting of four pound keys (##_##_##) was presented 32 times (null events). Each item was displayed for 5 s and followed by an inter-stimulus-interval of 2500 ms, resulting in a total of 24 min for this task, which was split in two functional runs á 12 min.

In both tasks stimulus categories were matched for problem size, distance, and parity. Order of stimulus categories and control items was randomized for each task. Participants responded with their dominant hand.

### Statistical analysis

Reaction times and error rates were evaluated and analyzed using software PASW statistics 17. For the number comparison task non-WD items were analyzed by a 2 × 2 repeated measures ANOVA with compatibility as within- and mathematics anxiety as between-subjects factor. For the number bisection task CB items were analyzed by a 2 × 2 ANOVA repeated measures ANOVA with multiplicativity as within- and mathematics anxiety as between-subjects factor.

### fMRI data acquisition and analysis

We acquired functional images as well as high resolution structural images on a 3T Philips Gyroscan NT scanner (Philips Medical System Inc., Maastricht, The Netherlands). For functional images 36 transversal slices were taken oriented parallel to the AC-PC line using a T2^*^-weighted gradient echo planar imaging (EPI) sequence (whole brain coverage, *TE* = 30 ms, *TR* = 2100 ms, flip angle 90°, slice thickness 3.0 mm with 0.6 mm gap, matrix 80 × 80, FOV 210 mm, in-plane resolution 2.6 × 2.6 mm). The TR was chosen such that it's ratio to each task's stimulus duration jittered the delay of stimulus onset relative to the TR. For structural images we used a T1-weighted 3D MPRAGE sequence (170 sagital slices, slice thickness = 1.2 mm, *TE* = 3.3 ms, TR 6.8 ms, TI delay 854 ms, FA 8°, FOV 256 × 256, matrix 256 × 256).

SPM5 (http://www.fil.ion.ucl.ac.uk/spm) standard procedures and templates were employed for analysis of functional images. The first five images of each session were discarded. Preprocessing steps were: (i) realignment and unwarping (Andersson et al., 2001), (ii) slice time correction, (iii) segmentation and normalization of structural images to MNI standard stereotactic space (iv) co-registration of functional and structural images (v) normalization of functional images using the parameters obtained in step (iii). To enhance activation detection, normalized functional images were resampled to isotropic 3 × 3 × 3 mm voxels and smoothed with a 6 mm Gaussian kernel.

For statistical analysis a two stage mixed effects model was applied. At first level the parameter estimates for each subject and item category were calculated by a canonical hemodynamic response function in the context of a GLM. Only correct responses were modeled. Reaction times as well as the six movement parameters were also included as regressors in the model. A high pass filter cut-off was set at 128 s. We corrected for autocorrelation by an AR(1) model (Friston et al., 2002).

The following first level contrasts were defined for the number comparison task: (i) all non-WD number comparison items vs. null events, (ii) incompatible items vs. compatible items. For the number bisection task we defined the following first-level contrasts: (i) all number bisection items vs. null events, (ii) non-multiplicative items vs. multiplicative items.

At second level group-based random-effects were evaluated. Task related activation areas were defined as regions showing higher BOLD response during the numerical task compared to null events. Task-related deactivation areas were defined as regions showing lower BOLD response during the numerical task compared to null events. HMAs and LMAs were compared using full factorial designs. All activation maps were thresholded at a voxel level threshold of *p* < 0.005 (uncorrected) and *k* > 50 voxels cluster size. The cluster-level FDR corrected *p*-value (threshold *p* < 0.05) was reported.

## Results

### Behavioral results

In order to replicate general numerical effects as described in the literature and to evaluate differences between individuals with high and LMA in task performance overall as well as in these numerical effects, 2 × 2 ANOVAs were conducted on RT and ER in the number comparison and number bisection task. Within-subjects factors were compatibility (compatible vs. incompatible items) in the analyses for the number comparison tasks, and multiplicativity (multiplicative vs. non-multiplicative) in the analyses for the number bisection task. The between-subjects factor was group (HMA vs. LMA). Descriptive statistics are summarized in Table 2.

**Table 2:** **Descriptive statistics (means ± SE) for reaction times (RT) and error rates (ER) in the numerical and non-numerical tasks in individuals with high (HMA) and low (LMA) mathematics anxiety**.

	**RT [ms]**		**ER [%]**	
	**HMA**	**LMA**	**HMA**	**LMA**
**NUMBER COMPARISON**				
Compatible	842.95 ± 34.49	836.09 ± 32.52	0.61 ± 0.31	1.11 ± 0.29
Incompatible	859.63 ± 34.25	869.15 ± 32.29	2.91 ± 0.68	3.15 ± 0.64
**NUMBER BISECTION**				
Multiplicative	2993.80 ± 148.70	2912.46 ± 140.18	9.71 ± 1.73	7.36 ± 1.64
Non-multiplicative	3061.23 ± 138.91	2941.87 ± 131.91	10.61 ± 2.13	10.69 ± 2.01
**GENERAL INTELLIGENCE**				
Mental rotation	12037.56 ± 2258.18	10864.82 ± 2237.65	48.13 ± 18.34	46.67 ± 18.79
Verbal reasoning	7803.39 ± 1628.29	7442.27 ± 1381.87	45.63 ± 17.50	35.56 ± 20.93

#### General numerical effects

##### Number comparison and the compatibility effect

Reaction times (RT) as well as error rates (ER) were significantly higher for incompatible compared to compatible items [compatibility effect; RT: *F*_(1, 32)_ = 18.33, *p* < 0.001; ER: *F*_(1, 32)_ = 25.80; *p* < 0.001] in number comparison replicating previous studies.

##### Number bisection and the multiplicativity effect

RT and ER did not differ between non-multiplicative and multiplicative items [multiplicativity effect; RT: *F*_(1, 32)_ = 1.95, *p* = 0.17; *F*_(1, 32)_ = 1.84, *p* = 0.18] in number bisection. This is in contrast to previous studies, although the descriptive differences pointed in the same direction as in previous studies (responses to non-multiplicative trials being descriptively slower and more error-prone than to multiplicative items).

#### Effects of mathematics anxiety

##### Main effect of mathematics anxiety

HMAs and LMAs did not differ in RT or ER in number comparison [RT: *F*_(1, 32)_ = 0.001, *p* = 0.98; ER: *F*_(1, 32)_ = 0.40, *p* = 0.53], or number bisection [RT: *F*_(1, 32)_ = 0.26, *p* = 0.61; ER: *F*_(1, 32)_ = 0.27; *p* = 0.60]. HMAs and LMAs did also not differ significantly in RT and accuracy on the non-numerical tasks (all |*t*| < 1.51, all *p* > 0.14).

##### Mathematics anxiety and the compatibility effect

There was no significant interaction of mathematics anxiety group with the compatibility effect on RT or ER in number comparison [RT: *F*_(1, 32)_ = 1.99, *p* = 0.17; ER: *F*_(1, 32)_ = 0.09; *p* = 0.77].

##### Mathematics anxiety and the multiplicativity effect

There was no significant interaction of mathematics anxiety group with the multiplicativity effect on reaction times and error rates in number bisection [RT: *F*_(1, 32)_ = 0.30, *p* = 0.59; ER: *F*_(1, 32)_ = 0.98, *p* = 0.32].

In sum, there were no differences between HMAs and LMAs in either the numerical tasks or in any of the numerical effects of interest. This suggests that the matching procedure has worked and has transferred to the numerical tasks assessed. Thus, any subsequent neurocognitive differences between the anxiety groups cannot be attributed to performance differences.

### Neuroimaging data

#### General numerical effects

In order to replicate general numerical effects on brain activation patterns as described in the literature, activation and deactivation areas were identified for each task. Furthermore, regions with stronger BOLD-response for incompatible as compared to compatible items were identified for the number comparison tasks and regions with stronger BOLD-response for non-multiplicative as compared to multiplicative items in the number bisection task.

##### Number comparison and the compatibility effect

**Figure 1:**
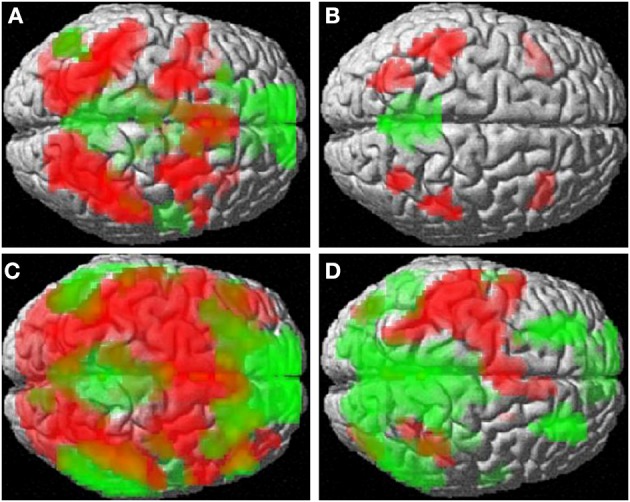
**Activation (red) and deactivation (green) patterns for (A) mental rotation, (B) verbal reasoning, (C) number bisection, (D) number comparison**.

Averaged across groups, number comparison led to pre-dominantly left-hemispheric activation of the parietal lobe, pre- and postcentral gyri and supplementary motor area (compare Figure 1). Deactivation areas are summarized in Table 3. Averaged across groups, no activation differences between compatible items and incompatible items (compatibility effect) were observed.

**Table 3:** **Default mode network**.

**Brain area**	**MNI-coordinates (mm)**			**Side**	**#voxels**	***T***	***p_FDR_***
	***x***	***y***	***z***				
**NUMBER COMPARISON**							
Precuneus/Cuneus/Calcarine g./ Post./mid. cingulate g./sup. occipital g.	27	−42	−15	Left/right	4400	9.87	< 0.001
Fusiform g./ Hippocampus/Parahippocampus	−24	−45	−15	Left	357	9.17	< 0.001
Mid./sup. temporal g./temporal pole	57	−3	−15	Right	75	6.79	0.002
Mid./sup. temporal g./temporal pole	−60	−9	−15	Left	87	6.12	0.008
Mid./sup. temporal g	−51	−51	18	Left	38	4.17	0.092
Mid./sup. frontal g.	−27	45	36	Left	235	6.47	0.004
Mid./sup. frontal g.	33	42	30	Right	174	5.55	0.024
Mid./sup. frontal g.	−18	66	3	Left	37	5.35	0.036
mPFC	9	54	−6		113	4.36	0.056
**NUMBER BISECTION**							
IPL/mid./sup. temporal g./ mid. occipital g./ fusiform g. Hippocampus/Parahippocampus/ Amygdala	39	−15	−6	Right	1775	11.42	<0.001
IPL/mid./sup. temporal g./ mid. occipital g.	−42	−57	21	Left	381	10.81	<0.001
mid. temporal g./ fusiform g./ Hippocampus/Parahippocampus/ Amygdala	−18	−6	21	Left	750	9.24	<0.001
mPFC/sup. frontal g./ ant. cingulate g./SMA	−6	57	3		2270	10.40	<0.001
Inf. frontal g.	54	33	3	Right	122	8.18	< 0.001
Precuneus/cuneus/calcarine g./ post./mid. cingulate g.	−9	−45	36		1233	8.93	< 0.001

*Clusters with significantly lower BOLD-response during the number comparison or number bisection tasks than during null events (= task-related deactivation clusters). P_uncorr_ < 0.001, k > 20 voxels, voxel-wise FDR-correction. G., gyrus; mid., middle; ant., anterior; post., posterior; sup., superior; inf., inferior; mPFC, medial prefrontal cortex; SMA, supplementary motor area; IPL, inferior parietal lobule*.

##### Number bisection and the multiplicativity effect

Averaged across groups, number bisection activated a large fronto-parieto-occipital network (compare Figure 1). Deactivation areas are summarized in Table 3. Non-multiplicative items as compared to multiplicative items (multiplicativity effect) led to stronger bilateral activation of the lateral occipital cortices [left: (−39, 84, 15), *T* = 5.30, *p*_FDR_ < 0.001, *k* = 91; right: (24, −87, 15), *T* = 6.31, *p*_FDR_ = 0.001, *k* = 71] and superior parietal lobules [left: (−21, −63, 54)], *T* = 4.71, *p*_FDR_ < 0.001, *k* = 45; right: (33, −54, 54), *T* = 4.45, *p*_FDR_ < 0.001, *k* = 62] involved in number magnitude processing (Moeller et al., 2009, 2010).

#### General non-numerical effects

##### Spatial reasoning

Averaged across groups, mental rotation lead to activation in a large fronto-parieto-occipital network, similar to the number bisection task (compare Figure 1). Deactivation areas included the mPFC [(−12, 39, 0), 657 voxels, *T* = 6.29, *p*_FDR_ < 0.001], precuneus [(−6, −54, 9), 945 voxels, *T* = 6.54, *p*_FDR_ < 0001], hippocampus [(−21, −12, −12), 51 voxels, *T* = 5.02, *p*_FDR_ < 0.05], the left angular gyrus [(−51, −69, 27), 104 voxels, *T* = 3.59, *p*_FDR_ < 0.001] and right middle temporal gyrus [(57, −6, −12), 107 voxels, *T* = 4.94, *p*_FDR_ < 0.001].

##### Verbal reasoning

Averaged across groups, the analogies task lead to activation in classical language areas including the inferior frontal gyri and inferior parietal lobules bilaterally (compare Figure 1). Deactivation was observed in the precuneus [(−6, −48, 24), 367 voxels, *T* = 5.44, *p*_FDR_ < 0.001].

#### Effects of mathematics anxiety.

In order to evaluate BOLD-response differences between HMAs and LMAs, the contrast images defined at first level (number comparison vs. null events; number bisection vs. null events) were compared between HMAs and LMAs at second level. Furthermore, the compatibility and multiplicativity contrasts as defined at first level (compatible vs. incompatible items; multiplicative vs. non-muliplicative items) were compared between HMAs and LMAs to evaluate possible interactions between compatibility or multiplicativity and mathematics anxiety.

##### Main effect of mathematics anxiety

**Figure 2:**
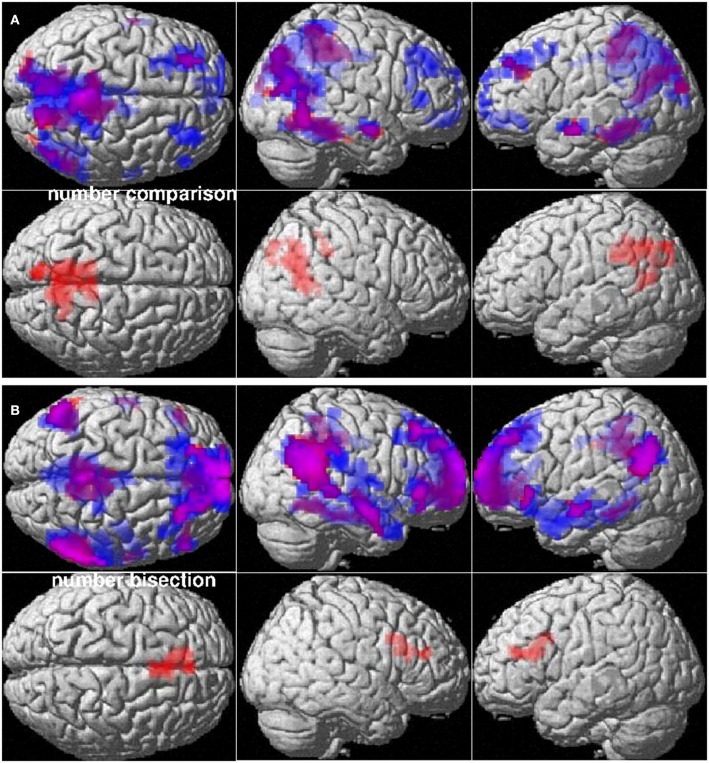
**Mathematics anxiety modulates task-related deactivation**. Upper panels: Deactivation patterns of individuals with high (pink) and low (blue) mathematics anxiety in **(A)** number comparison and **(B)** number bisection. Lower panels: Clusters with significantly stronger deactivation in individuals with low compared to individuals with high mathematics anxiety in **(A)** number comparison and **(B)** number bisection. Cluster-level FDR-corrected threshold: *p* < 0.05.

HMAs and LMAs did not differ in their BOLD response in areas activated by the number comparison or number bisection task. However, for both, number comparison and number bisection, deactivation within the task-related DMN was moderately stronger for LMAs compared to HMAs (Figure 2). For number comparison, a significant difference in BOLD-response between HMAs and LMAs was observed in the Precuneus [(−15, −39, 33), 541 voxels, *T* = 6.33, *p*_FDR_ < 0.001]. For number bisection, a significant difference in BOLD-response between HMAs and LMAs was observed in the anterior cingulate gyrus [(−6, 24, 18), 99 voxels, *T* = 4.51, *p*_FDR_ = 0.003]. No differences in BOLD response between HMAs and LMAs neither in activation nor deactivation were observed for spatial or verbal reasoning.

In order to demonstrate an interaction between type of task and group, eigenvariates were extracted from the Precuneus and the ACC in the two numerical and the two non-numerical tasks. Two 2 × 2 ANOVAs were performed over ACC eigenvariates with the within-subjects factor task (bisection vs. rotation; bisection vs. verbal) and the between-subjects factor group (HMA vs. LMA). A significant interaction between type of task (numerical vs. non-numerical) and mathematics anxiety group could be demonstrated for the ACC [rotation: *F*_(1, 31)_ = 13.95, *p* = 0.001; verbal: *F*_(1, 31)_ = 13.57, *p* = 0.001]. Furthermore, two 2 × 2 ANOVAs were performed over Precuneus eigenvariates with the within-subjects factor task (comparison vs. rotation; comparison vs. verbal) and the between-subjects factor group (HMA vs. LMA). A significant interaction between type of task (numerical vs. non-numerical) and mathematics anxiety group could be demonstrated for the Precuneus [rotation: *F*_(1, 31)_ = 4.32, *p* < 0.05; but verbal: *F*_(1, 31)_ = 2.22, *p* = 0.15].

##### Mathematics anxiety and the compatibility effect

**Figure 3:**
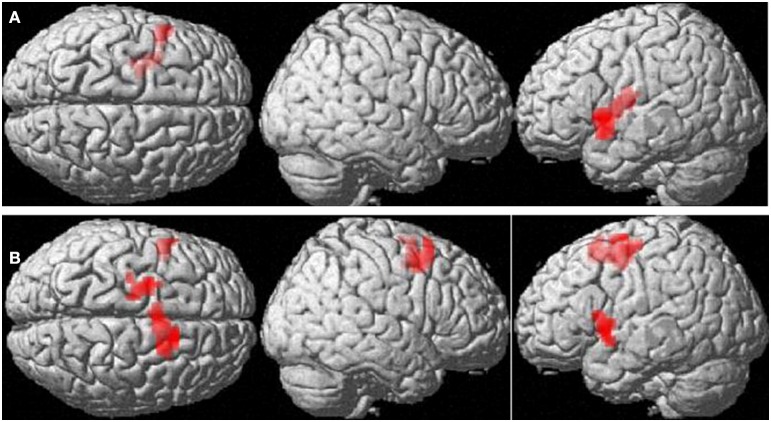
**Interactions with requirements on inhibitory functions. (A)** Clusters showing significantly stronger BOLD-response to incompatible compared to compatible items in participants with low mathematics anxiety. **(B)** Interaction between mathematics anxiety group and compatibility. Cluster-level FDR-corrected threshold: *p* < 0.05.

In the number comparison task mathematics anxiety group interacted with the compatibility effect in BOLD response in the left inferior frontal gyrus and Insula [(−54, 9, 3), 54 voxels, *T* = 4.72, *p*_FDR_ = 0.001], left dorsolateral prefrontal cortex [(−21, −9, 57), 53 voxels, *T* = 4.70, *p*_FDR_ < 0.05] and supplementary motor area [(12, 15, 54), 125 voxels, *T* = 4.68, *p*_FDR_ < 0.05] (compare Figure 3). As opposed to LMAs, HMAs failed to activate these regions, involved in inhibitory control, more strongly during incompatible items. Only LMAs did show a significant compatibility effect in the left inferior frontal gyrus and Insula [(−54, 9, 3), 131 voxels, *T* = 4.90, *p*_FDR_ = 0.001]. Consequently, the lack of identification of the compatibility effect across all participants was attributable to the lack of a compatibility effect in HMAs. These location of the result differed from previously reported results (Wood et al., 2006). However, the previous study did not include WD-items in their number comparison design, forcing participants to attend unit digits.

##### Mathematics anxiety and the multiplicativity effect

We did not observe an interaction of mathematics anxiety group with the multiplicativity effect in number bisection.

## Discussion

### General differences between high and low math anxiety participants in numerical tasks

In the present study, we compared individuals with HMAs and individuals with LMAs, who were matched for their mathematical performance, for their BOLD-response to two numerical tasks. We hypothesized that as has been demonstrated for general anxiety the increased emotional control required for HMAs to overcome their negative emotional response in comparison to LMAs would result in reduced deactivation of DMN areas involved in emotional and self-referential processing. This hypothesis was confirmed by results of the present study. In a simple number comparison task and a more complex number bisection task participants with HMAs show less deactivation of the DMN than participants with LMAs. Importantly, these differences were restricted to the mathematical tasks and no differences were observed in a spatial or a verbal reasoning task. As we did also not observe any performance differences between HMAs and LMAs in the non-numerical tasks from a general intelligence test, it seems unlikely that the deactivation differences between HMAs and LMAs were attributable to general differences in intelligence or working memory capacity. As DMN deactivation has been discussed as an indicator of processing efficiency, this result indicates reduced processing efficiency in HMAs compared to LMAs during mathematical cognition. HMAs simply need to put more effort into controlling their negative emotional response and reach comparable performance to LMAs. This finding is in good accord with previous finding of less efficient processing (Eysenck et al., 2007) and reduced DMN deactivation during rest (Simpson et al., 2001; Zald and Pardo, 2002) in individuals with other types of anxiety. This study is the first to demonstrate reduced deactivation of the DMN in highly anxious individuals during cognitive performance, even though their performance was matched to that of low anxious individuals. What is more, the study is also the first to demonstrate this effect for mathematics anxiety in particular.

### The neurocognitive basis: reduced default mode network deactivation to inhibit anxiety

We further hypothesized that this reduced deactivation indicates a preoccupation with the emotional value of the stimuli leading to a reduced capability to inhibit irrelevant information during the performance of mathematical tasks. We based this hypothesis on findings indicating that particularly the inhibitory functions of working memory are impaired in individuals with other types of anxiety (Derakshan and Eysenck, 2009; Wieser et al., 2009). This specific interpretation is corroborated by the within-task interactions of math anxiety with compatibility, which we will discuss in more detail.

In the number comparison task, unit-decade compatibility was varied as unit-decade incompatible items require the inhibition of unit digit information while unit-decade compatible items do not. In a compatible trial like 42_57, both the decade and the unit comparison lead to the same result, while in an incompatible trial like 47_62, the unit comparison of 7 and 2 led to a different result as the relevant decade comparison. Therefore, in incompatible trials the result of this unit comparison interferes with the overall comparison response and needs to be inhibited (for connectionist models specifying this inhibition mechanism see (Moeller et al., 2012; Huber et al., 2013, 2014). The neurocognitive data corroborate these model assumptions: Incompatible items led to stronger activation of right inferior frontal cortex, which has been discussed as the primary locus involved in inhibitory functions of working memory (Aron et al., 2004). Behaviorally, we were able to confirm longer reaction times and more errors for incompatible compared to compatible items (see Nuerk et al., 2001, 2011 for reviews).

Interestingly, the interaction between mathematics anxiety and compatibility was found in the inferior frontal cortex of the contralateral, i.e. the left hemisphere. In the left hemisphere, individuals with HMA failed to show enhanced BOLD-response of the inferior frontal cortex in response to incompatible items. This suggests less efficient processing, because task-irrelevant areas were activated to inhibit the math anxiety in HMA participants instead of inhibitory control areas relevant for the task.

### Anxiety did not affect activation of number-specific areas in adults

In contrast to above findings, mathematics anxiety however, did not affect task-related activation areas, in particular no area that has been related to number processing, like the IPS. This is in line with previous findings in adults (Lyons and Beilock, 2011, 2012) indicating that performance deficits in HMAs are not related to activity in number processing areas and consequently not attributable to lower mathematical skills. In these studies however, no direct comparison of the activation patterns of HMAs and LMAs was performed, but rather was the size of the performance deficit in HMAs correlated to BOLD-response. Our findings are however in contrast to a study in children (Young et al., 2012), indicating reduced activation of the IPS in HMAs. However, in neither of these previous studies was mathematical performance matched between HMAs and LMAs as in our study. Consequently, their HMA group may have included subjects with particularly poor mathematical skills and in particular HMAs that were not able to control their emotional reactions and overcome their performance deficits.

### No effects of math anxiety when no inhibitory control is required

Interestingly, we did not find mathematics anxiety to differentially affect BOLD-response to multiplicative and non-multiplicative items in the number bisection task. While the behavioral multiplicativity effect did not reach significance, the increased activation of the superior parietal lobule during processing of multiplicative items confirms that non-multiplicative items require number magnitude processing, while multiplicative items can be solved via arithmetic fact retrieval. Consequently, mathematic anxiety impairs processing efficiency equally in arithmetic operations involving the manipulation of number magnitudes and during arithmetic fact retrieval. However, multiplicative and non-multiplicative items do not differ in terms of requirements on inhibitory control in the number bisection task. Retrieval of multiplicative tables merely facilitates solving the task, but there is no need for inhibition.

Taken together with the results of the number comparison task, these results suggest further specificity of the neurocognitive impact of math anxiety, when performance is matched. Math anxiety induces relatively increased DMN processing in numerical task where inhibition is required. When only additional activation needs to be activate to facilitate task performance and no inhibition is necessary, there is no neurocognitive within-task effect of math anxiety. The most likely explanation is that lack of DMN activation reduction in HMA participants reflects an increased need to inhibit emotional processing. This inhibition process seems to be general and activation of those inhibition/cognitive control-related areas is particularly pronounced in those task conditions, which require cognitive inhibition as well.

### Summary

In summary we were able to demonstrate that like other types of anxiety, mathematics anxiety impairs deactivation of the DMN during mathematical tasks indicating reduced processing efficiency even or especially when no performance deficits are visible. Importantly, the effect becomes increasingly prominent with increasing requirements on inhibitory functions.

### Conflict of interest statement

The authors declare that the research was conducted in the absence of any commercial or financial relationships that could be construed as a potential conflict of interest.

## Supplementary material

Supplementary Figure 1 **Mathematics anxiety correlates negatively to mathematical abilities. PowerV, Verification Power; PowerP, Production Power; ^**^*p* < 0.01, ^***^*p* < 0.001**.
