# Supplementary figures and images for: Mathematics anxiety reduces default mode network deactivation in response to numerical tasks
Source: Front Hum Neurosci. 2015 Apr 21;9:202. doi: 10.3389/fnhum.2015.00202 (PMC4404831; doi:10.3389/fnhum.2015.00202)

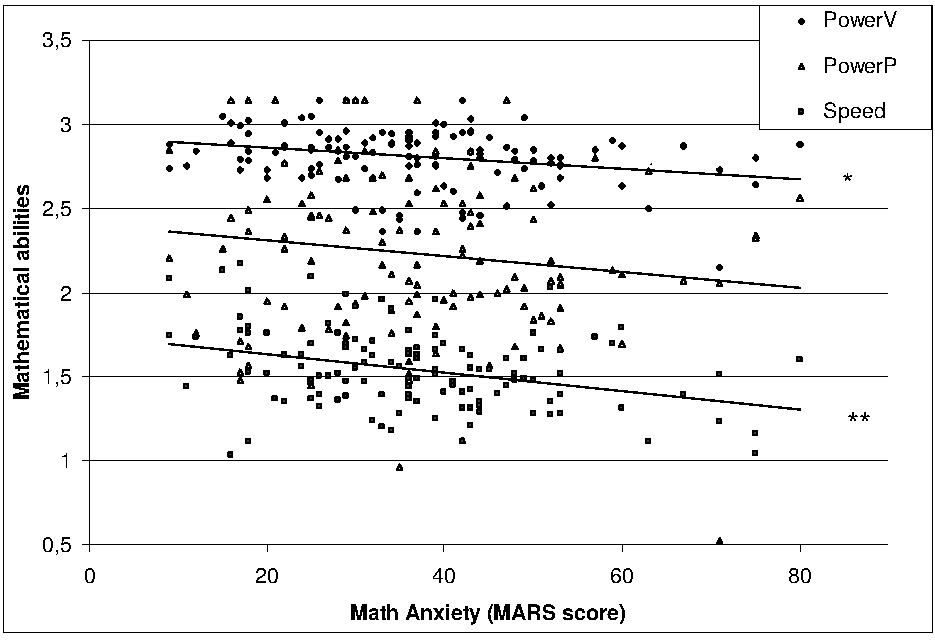

Supplement: Supplementary Figure 1 — Mathematics anxiety correlates negatively to mathematical abilities. PowerV, Verification Power; PowerP, Production Power; **p < 0.01, ***p < 0.001. [file Image1.TIF]
